# Advances in artificial intelligence models and algorithms in the field of optometry

**DOI:** 10.3389/fcell.2023.1170068

**Published:** 2023-04-28

**Authors:** Suyu Wang, Yuke Ji, Wen Bai, Yun Ji, Jiajun Li, Yujia Yao, Ziran Zhang, Qin Jiang, Keran Li

**Affiliations:** ^1^ Department of Ophthalmology, The Affiliated Eye Hospital of Nanjing Medical University, Nanjing, China; ^2^ The Fourth School of Clinical Medicine, Nanjing Medical University, Nanjing, China; ^3^ Affiliated Hospital of Shandong University of Traditional Chinese Medicine, Jinan, Shandong, China

**Keywords:** artificial intelligence, optometry, myopia, strabismus, amblyopia, corneal conus, artificial lens

## Abstract

The rapid development of computer science over the past few decades has led to unprecedented progress in the field of artificial intelligence (AI). Its wide application in ophthalmology, especially image processing and data analysis, is particularly extensive and its performance excellent. In recent years, AI has been increasingly applied in optometry with remarkable results. This review is a summary of the application progress of different AI models and algorithms used in optometry (for problems such as myopia, strabismus, amblyopia, keratoconus, and intraocular lens) and includes a discussion of the limitations and challenges associated with its application in this field.

## 1 Introduction

**FIGURE 1:**
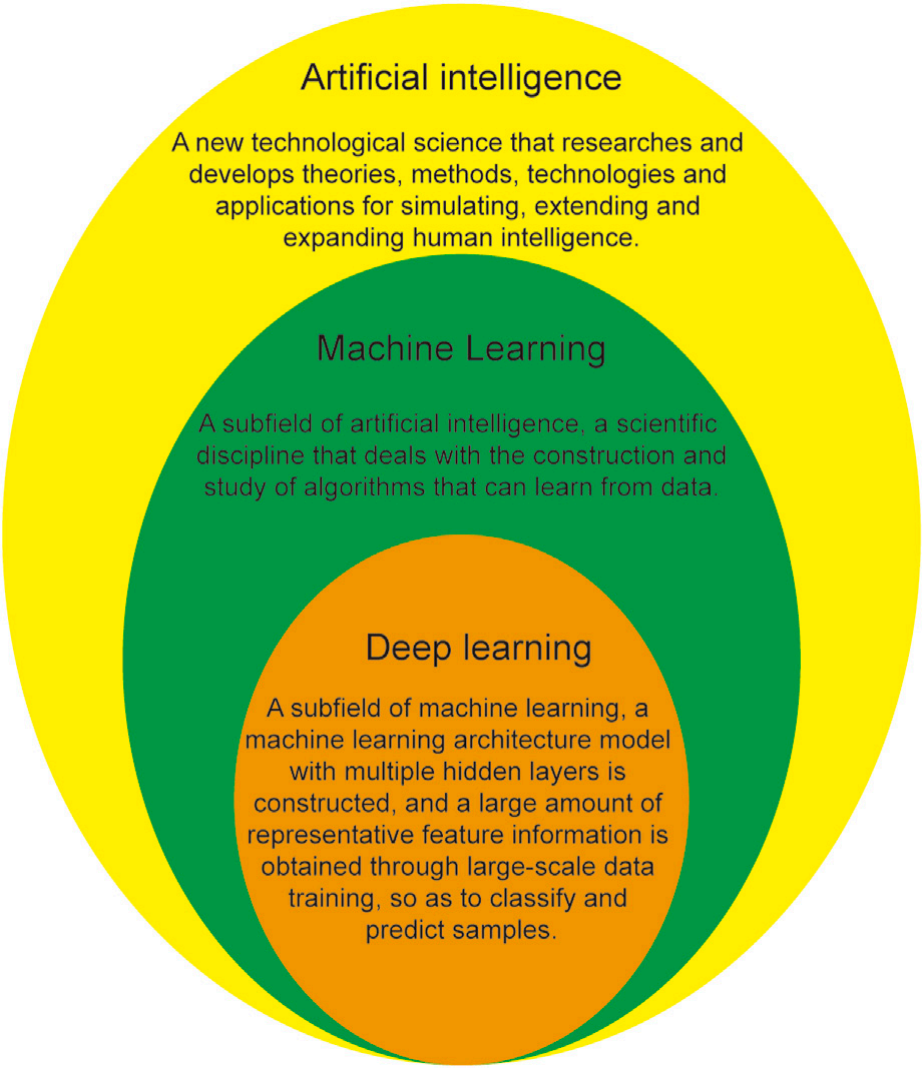
Relationship between AI, ML, and DL.

Artificial intelligence (AI) is a relatively new technology that endows machines with human behavior, thinking, and emotional abilities and can liberate human beings from tedious physical and mental labor and assist in the production and development of fields such as the economy, culture, and social life. AI, as a subfield of computer science, simulates human intelligence using algorithms that are developed using computers to engage in human work (Nuzzi et al., 2021). Machine learning (ML) is a research field of AI, which is a technology that allows computer systems to learn automatically from data and improve performance. Deep learning (DL) is a research field of ML, which is a representation learning algorithm based on artificial neural network. The relationship between AI, ML, and DL is shown in Figure 1. Current, commonly used AI algorithms include ML, DL, artificial neural networks, deep neural networks (DNNs), convolutional neural networks (CNNs), and migration learning. Over the past few years, the great development of computer science and technology has led to accelerated evolution in the field of AI, accelerating its application in medicine, especially ophthalmology. The first ophthalmic AI device, IDx-DR, was approved for listing with landmark significance on 11 April 2018, opening a new chapter on the combination of AI with ophthalmology. Since then, the application of AI in the ophthalmology has entered a new stage of development, leading to a series of satisfactory research results in the diagnosis, classification, recognition, and screening of ophthalmic diseases, such as diabetic retinopathy (Deepa et al., 2022; Hardas et al., 2022; Zhang et al., 2022), age-related macular degeneration (Glaret Subin and Muthukannan, 2022; Sotoudeh-Paima et al., 2022; Wang et al., 2022), retinopathy of prematurity (Coyner et al., 2022; Li et al., 2022; Wu et al., 2022), glaucoma (Dong et al., 2022; Li et al., 2022; Xiong et al., 2022), and retinal vein occlusion (Miao et al., 2022; Ren et al., 2022; Zhang et al., 2022).

**FIGURE 2:**
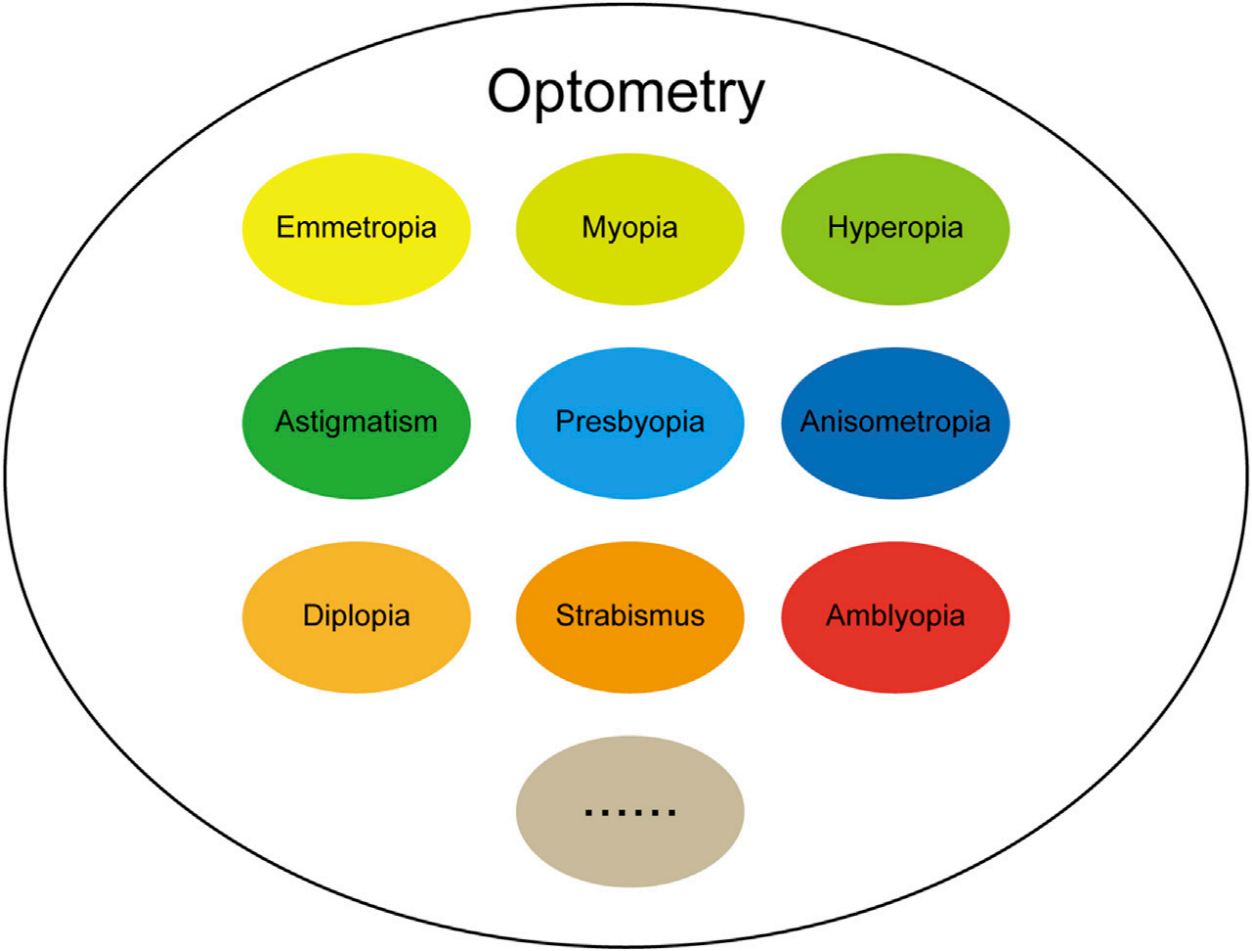
The basic composition of the field of optometry.

The term optometry originated in the ancient Greek words optos (“see”) and metron (“measure”), indicating that it is closely related to “eyes” and “vision.” At the beginning of the 20th century, visual optics was defined as “studying the philosophy of light and vision,” and included a deep understanding of the connotation of the relationship between “light” and “vision”; By the middle of the 20th century, people understood optical vision as “the art of determining the visual state of normal people or correcting the abnormal state through glasses,” and the understanding and correction of vision became more specific. After hundreds of years of evolution and development, optometry has since developed rapidly. In terms of composition, the field of optometry mainly includes emmetropia, myopia, hyperopia, presbyopia, astigmatism, anisometropia, strabismus, amblyopia and so on, as shown in Figure 2. The continuous increase in the application of AI in the ophthalmology in recent years has achieved many remarkable research results. Here, we aimed to review the recent research results of using AI in the field of ophthalmic optometry over the past few years, with the challenges and limitations of AI in optometry applications discussed.

## 2 Application of AI models and algorithms in the field of optometry

**FIGURE 3:**
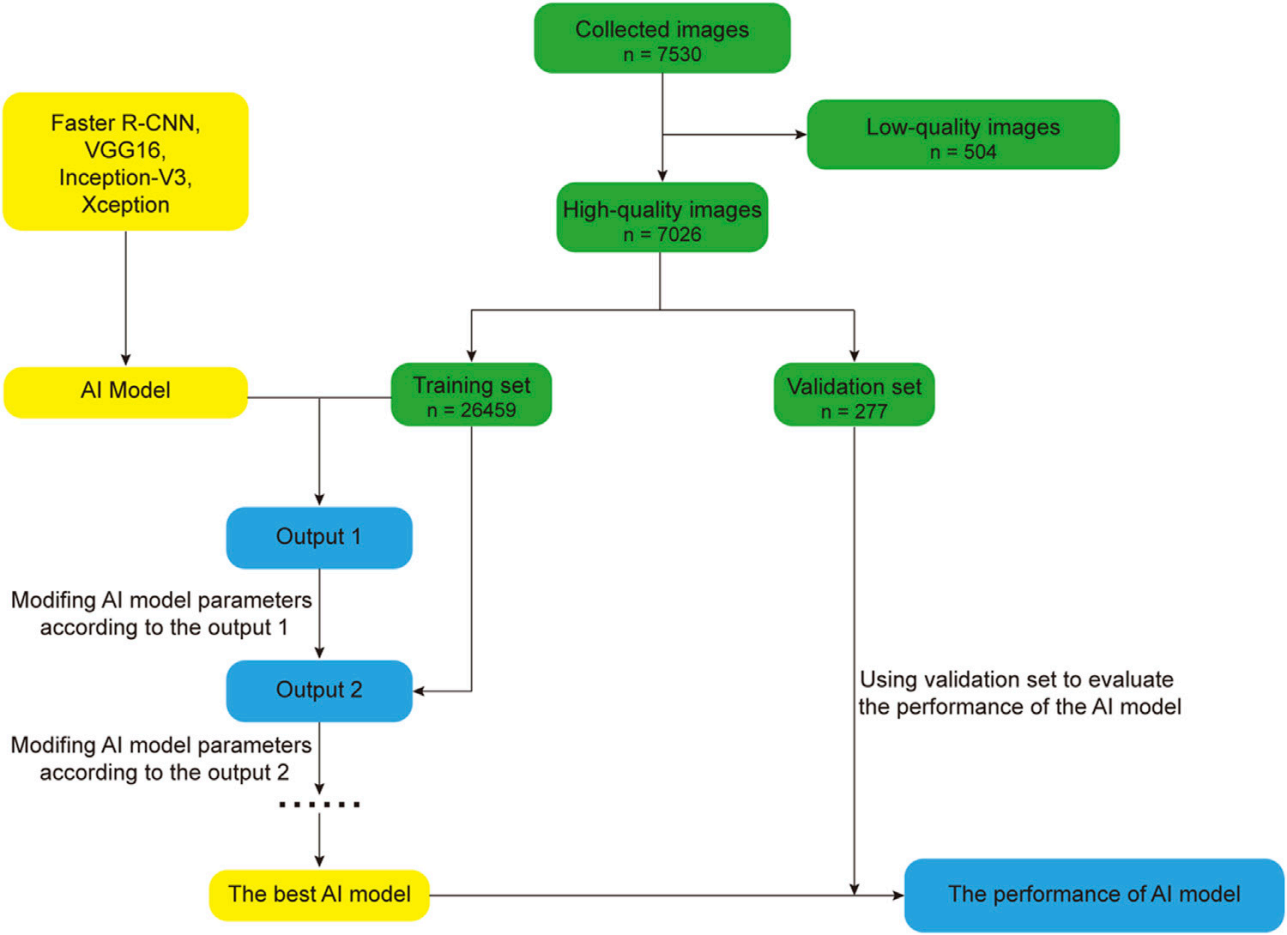
Basic flow chart illustrating AI model research.

In this section, we mainly review the research progress of AI in the field of optometry in the past 5 years. AI has carried out a lot of research in the field of optometry, especially in the diagnosis, screening and treatment of diseases. In recent years, with the continuous development and improvement of AI technology, the research of AI in the field of optics has become more in-depth and extensive. In order to better describe the basic flow of AI research, we take the DL model in Zheng et al. (2021) as an example, the basic flow of AI model research is illustrated in Figure 3. The process of developing an AI model involves deleting low-quality images and randomly dividing the remaining high-quality images into training and verification sets, with which the training set is optimized to obtain the best possible AI model and the performance is verified using the verification set.

### 2.1 Application of AI models and algorithms in myopia

Myopia is a type of ametropia in which parallel light is focused on the front of the retina through the intraocular refractive medium and thus a clear image is not formed on the retina. The problem occurs mainly during childhood and early adulthood (Morgan et al., 2018). Although there is still much to learn about the etiology of myopia, the general consensus is that genetic, environmental, and biochemical variables play a role in the development of myopia. Myopia in children is mostly caused by a decline in outdoor playtime and an increase in time spent staring at screens. The mechanism of myopia progression mainly includes: 1) accommodative lag, 2) retinal peripheral defocus theory, 3) scleral thinning and ocular axial lengthening caused by extracellular matrix remodeling, 4) changes in the level of retinal nerve growth factor and inflammatory factors, *etc.* According to recent studies, alterations in the choroid’s thickness and function are also variables in the evolution of myopia. The choroid may operate as a barrier to the diffusion of endogenous growth hormones that encourage axial elongation. Adults over 50 can also develop nuclear myopia due to cataracts (Amirsolaimani et al., 2017; Bullimore and Brennan, 2019; Wong et al., 2021). The issue is thus a public health problem of widespread concern. High and pathological myopia can significantly increase the incidence of retinal detachment, myopic macular degeneration, macular choroidal neovascularization, and other diseases. According to statistics, more than 150 million people worldwide suffer from moderate to severe visual impairment due to uncorrected ametropia (Baird et al., 2020; Blindness G.B.D, 2021). At present, the main treatment methods are drug therapy (such as atropine eye drops), instrumental correction (such as frame glasses and keratoscopes), and surgical treatment (such as femtosecond pulsed or excimer lasers) (Li and Yam, 2019; Weiss and Park, 2019; Tsai et al., 2021). However, the complex causes and the large number of people affected renders large-scale screening and stratified analysis of myopia difficult, and the serious complications that are associated with the problem are generally not detected early enough for suitable intervention. It is therefore important that high-risk groups for high myopia are accurately identified and treated in a timely and effective manner to delay the progression of the disease. The development of AI for medical use has led to remarkable results in myopia prediction, diagnosis, screening, follow-up, and treatment (Gunasekeran et al., 2021). It can achieve effective data management and analysis, deeply excavating the inherent mechanisms by which myopia develops, and the big data storage function associated with AI is conducive to the accumulation of numerous individual experiences, thus playing an important auxiliary role in diagnosis and classification.

One development in the use of AI to diagnose myopia was made by Varadarajan et al. (2018), who constructed a DL model based on a residual network and a soft-attention layer and used it to analyze 226 870 fundus images. The model can predict spherical diopters, cylindrical diopters, and equivalent spherical equivalents by analyzing millions of parameters such as picture pixel values to judge the ametropia situation. A total of 39 757 fundus images were used to validate the model. The results showed average absolute errors of 0.56 D and 0.91 D, respectively, for the two data sets, realizing a technical leap in accurately predicting refractive errors from retinal fundus images. Lin et al. (2018) established an AI model that can predict myopia in children on the basis of a random forest algorithm using refractive data for 132 457 children from the electronic medical record database at eight eye centers, which were applied to training and verification. The model predicts myopia in children under 18 years of age by analyzing data such as age, spherical equivalent, and the annual myopia progression rate. The results showed an AUC between 0.940 and 0.985 for high myopia within 3 years, 0.856 and 0.901 within 5 years, and 0.801 and 0.837 within 8 years, indicating that the model can accurately predict the incidence of high myopia in school-age children at specific points in the future. In a studying the prediction of axial myopia, Tang et al. (2020) used six different ML algorithms to build an AI model that can predict axial length in children and optimized the AI model using cross-sectional datasets. The axial length for the children was predicted by analyzing variables such as sex, age, central corneal thickness, spherical equivalent error, K-means, and the black-and-white corneal diameter for 1,011 myopic children aged 6–18 years old. The results indicated that robust linear regression model had the best prediction result for eye-axis length, with an R 2 of 0.87, proving that the algorithm could be used to estimate the physiological components of eye axis growth and provide data support for separating non-physiological components from eye axis elongation for other therapeutic methods. To better predict adolescent myopia, Yang et al. (2020) used a support vector machine to establish a prediction model for juvenile myopia, using data from 3,112 pupils (including heredity, eye habit, environment, and diet) and constructed a dataset for training and testing the model using univariate and multivariate correlation analyses. The results showed an accuracy, specificity, sensitivity, and AUC of 0.93, 0.94, 0.94, and 0.98, respectively, for predicting myopia, proving that the model can comprehensively analyze several causes of myopia and can be used to help formulate myopia prevention and control policies. These studies indicate that AI has opened up a novel prediction model for myopia prediction through image and related data analysis that has high accuracy, is feasible for clinical application, and provides new ideas for myopia prevention and control. Foo et al. (2023) developed a deep learning system to identify children at risk of developing high myopia. In this study three distinct algorithms were derived (image, clinical and mix models) to predict the development of high myopia in adolescents after 5 years. 7,456 baseline fundus images were used for training and verification, 821 images with clinical data for external validation. Results showed that this DLS achieved a high accuracy with AUC all above 0.90, and can prevent the progression and complications of myopia in adulthood, help ophthalmologists to make clinical decisions.

Through analyzing eye images, AI can thus assist in the diagnosis and classification of myopia, improving the diagnostic efficiency and aiding ophthalmologists in screening for large-scale myopia while also facilitating the long-term follow-up of patients with high myopia, reducing the heavy burden caused by visual impairment and even blindness that can result from myopia. Sogawa et al. (2020) constructed several models using different DL algorithms (VGG16, VGG19, DenseNet121, InceptionV3 and ResNet50) and used them to analyze 910 eye optical coherence tomography (OCT) images. The experimental results showed that the AUC, sensitivity, and specificity for the DL model were 0.970, 0.906, and 0.942; the average correct classification rate for high myopia, myopic choroidal neovascularization, and retinoschisis images of 0.889 shows the feasibility of using these algorithm models in screening for single diseases and provides support for preventing vision loss in patients with myopic macular degeneration. To assist diagnosis of myopia in the clinical, Yang et al. (2020) constructed an AI diagnosis model using deep convolutional neural networks (DCNNs) and the VGG-Face algorithm. In this study, the eye appearance images of 2,350 children aged 6–18 years were collected from three angles; front, side, 45° anterior side, and spherical equivalent refraction was used to determine the refraction state of each eye from the images. The AUC, sensitivity, and specificity of the model for the diagnosis of myopia were 0.9270, 0.8113, and 0.8642, respectively, after training and verification, rendering vision screening possible without the need for examination, thus providing a more convenient method for routine vision screening. Hemelings et al. (2021) used the CNN to construct a DL model that can diagnose pathological myopia and used 1,200 color fundus images for the model training and testing. The final results showed that the AUC of the model for the diagnosis of pathological myopia was 0.9867. Li et al. (2023) collected 1,200 retinal fundus images to train a novel deep learning model on the basis of MyopiaDETR algorithm. This model using 2D fundus images as input, which can diagnose and discriminate different kinds of myopia such as normal myopia (NM), high myopia (HM) and pathological myopia (PM) through the analysis of the images. Besides, it has significant advantages over the traditional algorithms in terms of the accuracy and speed of the diagnosis. The results showed excellent localization and classification performance in the diagnosis of PM, reaching AP50 of 0.8632. Li et al. (2022) collected 412 OCT macular images of patients with high myopia and constructed an AI model based on the InceptionResnetV2 algorithm to identify four visual threats: retinoschisis, macular hole, retinal detachment, and pathological myopic choroidal neovascularization. The results showed an AUC of between 0.961 and 0.999 for the model, with both sensitivity and specificity reaching >0.90. These results show that AI is particularly accurate in diagnosing myopia and its complications and indicate that it can play an important role in large-scale myopia screening.

Refractive surgery, which can be divided into corneal refractive surgery and intraocular refractive surgery, is used to correct refractive errors in adult patients with stable myopia. At present, corneal refractive surgery comprises laser epithelial keratomileusis (LASEK), laser *in situ* keratomileusis (LASIK), and small-incision lens extraction (SMILE). Intraocular surgery includes lens implantation and cataract surgery. Some progress has also been made in the application of AI to preoperative screening and surgical planning for refractive surgery. Xie et al. (2020) constructed a DL model based on the InceptionResNetV2 algorithm used 6,465 corneal tomography images (including axial curvature, anterior corneal topography, posterior corneal topography, and corneal thickness) to train and test the model to screen potentially suitable patients for refractive surgery, with a screening accuracy of 0.947. Yoo et al. (2020) developed an ML model based on a multiclass XGBoost model that can select the best refractive surgery for patients with myopia. The algorithm can automatically extract 80 features from corneal topography and convert the numbers from the image into textual data. Eye examination data were collected from 18 480 myopic patients who planned to undergo refractive surgery and divided into the LASEK, LASIK, SMILE, and contraindication groups. The results showed accuracies of 0.810 and 0.789 for the internal and external verification datasets, respectively, indicating that it can synthesize ophthalmic data and select the most suitable operation plan at expert level. Wan et al. (2023) constructed a Deep learning model on basic of Resnet50 and XG Boost algorithms, aiming to Predict the early postoperative visual acuity after small-incision lenticule extraction surgery. In this study, 10,176 laser scanning images from the surgical videos were collected for training, and patients were classified by good or poor recovery. The results turned out with the accuracy of 0.96, AUC value of the DL model was 0.962–0.998. This model enables accurate prediction of early postoperative vision and complications only through surgical videos and pictures, which has an important influence on the application of AI in refractive surgery.

Contact lens Contact lens is a common adjuvant therapeutic tool in the field of optometry, includes rigid contact lens, soft contact lens and Orthokeratology (OK). Orthokeratology is a crucial component of clinical myopia management since it is a successful myopia control strategy. Nowadays, Orthokeratology (OK) is second only to muscarinic antagonists in terms of effectiveness in reducing childhood myopia. AI-assisted contact lens therapy is gradually becoming popular. In order to predict the curvature of orthokeratology lens, Fan et al. (2022) construct a machine learning model based on Linear Regression (Robust), Support Vector Machines (linear), Bagged Trees, Gaussian processes algorithms. Using sex, age, horizontal visible iris diameter (HVID), spherical refraction (SER), anterior chamber depth (ACD), axial length (AL) *etc.*, of 1,271 patients with myopia as input variables, to estimate the alignment curve (AC) curvature of orthokeratology lens. Results indicated that the linear SVM and Gaussian process machine learning models achieves best performance, the R-squared values for the output AC1K1, AC1K2 and AC2K1 values were 0.91, 0.84, and 0.73. Prediction of orthokeratology lens curvature based on the ML model can reduce the number of lens trials, improve efficiency and accuracy, and reduce the probability of cross-infection caused by the test lens. By analyzing the clinical data of 1,037 Chinese myopic adolescents, Fan et al. (2021), developed a ML model on basic of Support vector machines (SVMs), Gaussian processes, Linear Regression (Robust) algorithms. This model is able to predict the return zone depth (RZD) and landing zone angle (LZA) of four quadrants of corneal refractive therapy (CRT) lenses under different combinations of age, sex and ocular parameters. Results showed that this model achieved higher accuracy, and is easier to use and faster to implement compared to the traditional sliding card method. To predict the treatment effect of orthokeratology, Fang et al. (2023) developed a ML model based on Logistic least absolute shrinkage and selection operator (LASSO) regression algorithm. The study collected the ocular parameters and clinical characteristics of 91 patients undergoing ortho-k treatment. It turned out that factors such as, lens wearing time, age, axial length, outdoor activity time, and white-to-white distance were strongly associated with treatment effects, with AUC values 0.949 and C-statistic of the predictive model was 0.821. This demonstrates how the ML model-based prediction of contact lens efficacy can help clinicians make clinical judgments and select more suitable treatment alternatives for patients. The above studies are summarized in Table 1.

**TABLE 1:** Application summary of different AI models and algorithms used in myopia.

Authors	Task	Sample size	AI algorithms	Output
Varadarajan et al. (2018)	Prediction	266627 images	Residual network, Soft-attention layer	Average absolute error of two datasets = 0.56 D, 0.91 D
Lin et al. (2018)	Prediction	132 457 individuals	Random forest	AUC for 3 years = 0.940–0.985
				AUC for 5 years = 0.856–0.901
				AUC for 8 years = 0.801–0.837
Tang et al. (2020)	Prediction	1,011 individuals	Linear Regression (linear)	R2 of robust linear expression model = 0.87
			Linear Regression (Robust)	
			SVM (linear)	
			SVM (Quadratic)	
			SVM (Cubic)	
			Bagged Trees	
Yang et al. (2020)	Prediction	3,112 individuals	SVM	Accuracy = 0.93
				Specificity = 0.94
				Sensitivity = 0.94
				AUC = 0.98
Foo et al. (2023)	Prediction	7,456 images	image, clinical and mix (image + clinical) models	Image models
				AUC of Primary dataset = 0.93–0.95
				AUC of Test dataset = 0.91–0.93
				Clinical models
				AUC of Primary dataset = 0.90–0.97
				AUC of Test dataset = 0.93–0.94
				Mixed (image + clinical) models: AUC of Primary dataset = 0.97
				Test dataset = 0.97–0.98
Sogawa et al. (2020)	Classification	910 images	VGG16	AUC = 0.970
			VGG19	Sensitivity = 0.906
			DenseNet121	Specificity = 0.942
			InceptionV3ResNet50	Accuracy = 0.889
Yang et al. (2020)	Diagnosis	2,350 individuals	DCNN	AUC = 0.9270, sensitivity = 0.8113
			VGG-Face	specificity = 0.8642
Hemelings et al. (2021)	Diagnosis	1,200 images	CNN	AUC = 0.9867
Li et al. (2023)	Diagnosis	1,200 images	MyopiaDETR	A PAP50 = 0.8632
Li et al. (2022)	Classification	412 images	InceptionResnetV2	AUC = 0.961–0.999
				Sensitivity >0.90, Specificity >0.90
Xie et al. (2020)	Screening	6,465 images	InceptionResNetV2	Accuracy = 0.947
Yoo et al. (2020)	Surgery	18,480 individuals	Multiclass XGBoost	Accuracy of internal validation dataset = 0.81
				Accuracy of external validation datasets = 0.789
Wan et al. (2023)	Prediction	10176 images	Resnet50	Accuracy = 0.96
			XG Boost	AUC = 0.962–0.998
Fan et al. (2021)	Prediction	1,271 individuals	Linear Regression (Robust)	R-squared values for the output AC1K1, AC1K2 and AC2K1 values = 0.91, 0.84, 0.73
			SVM (linear)	
			Bagged Trees	
			Gaussian processes	
Fan et al. (2021)	Prediction	1,037 individuals	SVM (SVMs)	R values for the nasal, temporal, superior and inferior LZA = 0.843, 0.693, 0.866, 0.762, RZD = 0.970, 0.964, 0.975, 0.964
			Gaussian processes	
			Linear Regression (Robust)	
Fang et al. (2023)	Prediction	91 individuals	Logistic least absolute shrinkage and selection operator (LASSO) regression	AUC = 0.949
				95%CI:0.815, 0.827

Myopia as a common refractive problem exists widely in adolescents and adults. Myopia tends to progress rapidly in adolescence. Variable degrees of fundus changes will also be present in individuals with high myopic in addition to vision loss, floaters, and flash sensations. The risk of retinal detachment, hiatus, fundus hemorrhage, and neovascularization is significantly higher than it is in healthy individuals. Therefore, early prediction and diagnosis of different types of myopia are of great significance for myopia treatment and prevention of complications. The prediction, classification diagnosis and auxiliary treatment of myopia based on artificial intelligence can greatly improve the diagnosis and treatment efficiency and accuracy of clinicians, and play an important role in the large-scale screening of myopia.

### 2.2 Application of AI models and algorithms in strabismus

Strabismus refers to any clinical phenomenon of visual axis deviation that can be caused by binocular abnormalities, neuromuscular abnormalities in eye movement control, or various other mechanical limitations (Sousa de Almeida et al., 2015). Strabismus can be divided into different types according to fusion state, eye movement and fixation, eye position, and age of occurrence (Castanes, 2003; Mojon-Azzi et al., 2011), and is commonly associated with visual development in children. Studies have shown that a prevalence of is 2%–4% for strabismus in children worldwide, which is significantly higher than observed in adults (Chia et al., 2010). One of the main issues with strabismus is that it can lead to abnormal visual functions such as strabismic amblyopia and seriously endanger the physical and mental health of infants and children, rendering timely diagnosis and treatment particularly important (Kelkar et al., 2015; Debert et al., 2016). At present, the common examination methods for strabismus in clinics include masking and cover-uncover tests, alternate cover tests, prism and cover tests, corneal reflection methods, synoptophore examinations, diagnostic strabismus tests, and eye movement traction tests (Chia et al., 2007; Wang et al., 2018; Yoo et al., 2019). Traditional strabismus diagnosis methods usually require manual examination by ophthalmologists, which is time-consuming and labor-intensive with subjective results. The application of AI technology in strabismus amblyopia has achieved muchand is thus expected to improve the current state of diagnosis and treatment for strabismus amblyopia.

Kang et al. (2022) constructed a DL model based on the U-Net network that can segment the cornea and scleral limbus and then classify the segmented eye region to realize automatic strabismus detection using 828 gaze photographs of strabismus patients with different eye positions used to train and verify the model. After verification, an accuracy of 0.9984 was obtained for corneal segmentation using the model, with a sensitivity of 0.9747, specificity of 0.9990, diameter similarity coefficient (DSC) of 0.9688, while the accuracy of limbal segmentation was 0.9992, with a sensitivity of 0.9563, specificity of 0.9996, and DSC of 0.9571. To develop a DL system that can assist in diagnosing strabismus, Mao et al. constructed a system based on InceptionResNetV2 using 5,797 corneal light reflection photos to develop, train, and verify the system (Mao et al., 2021). The system diagnoses strabismus by identifying different gaze states in reflective corneal photos. After training and testing, the experimental results showed sensitivity, specificity, and AUC of 0.991, 0.983 and 0.998 respectively, for the system. Another app that was developed by de de Figueiredo et al. (2021) based on the Resnet50 neural network can diagnose strabismus by identifying the different gaze positions of patients. The app was developed using gaze photos from 110 patients and the overall accuracy in the diagnosis of strabismus was between 0.42 and 0.92, with precision between 0.28 and 0.84. The above studies show that the AI model performs well in the auxiliary diagnosis of strabismus and has the potential for clinical application, where it may improve the accuracy of strabismus diagnosis and reduce the work pressure of clinicians.

Zheng et al. (2021) used a convolutional neural network and three deep convolution neural networks (Faster R-CNN, VGG16, Inception-V3, and Xception) to construct a DL model that can detect strabismus through the gaze of children using 7,530 primary gaze photos to develop, train, and verify the model. After external verification, a sensitivity of 0.940 was with a specificity of 0.993, AUC of 0.990, and accuracy of 0.950, for the model, which is better than that acquired by clinicians. Chen et al. (2018) constructed a DL model that can recognize strabismus using six different convolution neural networks (AlexNet, VGG-S, VGG-M, VGG-16, VGG-F, VGG-19). They collected gaze deviation images from 42 subjects and used them to train and verify the model. The results indicated that VGG-S had the best performance in recognizing strabismus, with a specificity of 0.960 and sensitivity of 0.941. Huang et al. (2022) constructed a strabismus screening and classification method based on the ResNet-12 network and used positive facial images from 60 subjects to train and test. This method identifies eye position in frontal facial images to diagnose strabismus and resulted in accuracy, sensitivity, and specificity for screening and classification values of 0.805, 0.768, and 0.842, respectively. To better assist in strabismus screening, Huang et al. (2021) constructed a DL model for strabismus screening based on the convolution neural network with 60 frontal facial images for training and verification. The experimental results showed that sample mean and standard deviation values for normal images of 1.073 ± 0.014 and 0.039, respectively, while those for strabismus images were 1.924 ± 0.169 and 0.472, respectively. The results of the above AI model in strabismus screening and recognition indicate that AI will likely be applied to strabismus screening in the future. The development of remote diagnosis methods for strabismus also overcomes limitations surrounding spatial distance, which is also significant for early detection and treatment in ophthalmopathy.

Liu et al. (2019) developed a DL model based on a support vector machine that can predict the time it will take for a patient to achieve visual function following strabismus surgery from the deviation angle of the eye position 1 day and 6 months after the operation. In this study, using the surgical data of 132 patients to train and test the model and a prediction accuracy of 0.821 was achieved. To aid patients with strabismus in selecting the best surgical treatment strategy, Almeida et al. (2015) proposed an AI method based on support vector regression, with the clinical data of 88 strabismus patients used to train and verify the method. This method can be used to decide the best surgical treatment strategies for strabismus patients according to the deviation degree, deviation type, visual acuity data, diopter, and fundus examination data of strabismus patients. Finally, the results showed that the average error in the proposed surgical treatment strategy was 0.5 mm for recoil and 0.7 for resection for medial rectus surgery while the mean error was 0.6 for recoil and 0.8 for resection in lateral rectus surgery, indicating that this method is feasible for use in planning strabismus surgery. Lou et al. (2022) constructed a novel recurrent residual CNN with global attention gate based on GAR2U-Net to automatically evaluate the Inferior oblique overaction (IOOA). This study included 106 eyes of 72 consecutive patients, and the height difference between the inferior corneal limbus of both eyes were measured. The results showed significant correlations measurements and clinical gradings. The new method allows for objective, accurate and reproducible IOOA measurements and has obvious advantages such as low cost, easy acquisition and wide measurement range compared with conventional methods. The above studies are summarized in Table 2.

**TABLE 2:** Application summary of different AI models and algorithms used for strabismus.

Authors	Task	Sample size	AI algorithms	Output
Kang et al. (2022)	Diagnosis	828 images	U-Net	Accuracy = 0.9984
				Sensitivity = 0.9747
				Specificity = 0.9990
				DSC = 0.9688
Mao et al. (2021)	Diagnosis	5,797 images	InceptionResNetV2	Sensitivity = 0.991
				Specificity = 0.983
				AUC = 0.998
de Figueiredo et al. (2021)	Diagnosis	110 individuals	Resnet50	Accuracy = 0.42–0.92
				Precision = 0.28–0.84
Zheng et al. (2021)	Detection	7,530 images	Faster R-CNN	Sensitivity = 0.940
			VGG16	Specificity = 0.993
			Inception-V3,Xception	AUC = 0.990
				Accuracy = 0.950
Chen et al. (2018)	Detection	42 individuals	AlexNet	Specificity = 0.960
			VGG-F	
			VGG-M	
			VGG-S	Sensitivity = 0.941
			VGG-16	
			VGG-19	
Huang et al. (2022)	Detection	60 individuals	ResNet-12	Accuracy = 0.805
				Sensitivity = 0.768
				Specificity = 0.842
Huang et al. (2021)	Detection	60 images	CNN	The sample mean and standard deviation of normal images = 1.073 ± 0.014, 0.039
				The sample mean and standard deviation of strabismus images = 1.924 ± 0.169, 0.472
Liu et al. (2019)	Prediction	132 individuals	SVM	Accuracy = 0.821
Almeida et al. (2015)	Prediction	88 individuals	Support Vector Regression	The average error for recoil = 0.5 mm
				The average error for resection = 0.7 mm
				The mean error for recoil = 0.6 mm
				The mean error for resection = 0.8 mm
Lou et al. (2022)	Prediction	106 eyes	GAR2U-Net	Kendall’s tau: 0.721; 95% confidence interval: 0.652 to 0.779; *p* < 0.001

Strabismus as a common eye disease, if not timely diagnosis and treatment, may lead to significant vision loss or even blindness. More importantly, strabismus will bring serious psychological burden to patients, resulting in many adverse consequences. Therefore, it is very important for timely diagnosis and treatment of strabismus. The above AI studies show that AI can play an important role in the diagnosis of strabismus. It can not only diagnose strabismus without ophthalmologist, but also significantly reduce the cost of diagnosis.

### 2.3 Application of AI models and algorithms in keratoconus

Keratoconus (KC) is a non-inflammatory corneal disease characterized by thinning of the corneal stroma, anterior protrusion, and irregular astigmatism. Thinning occurs in or near the center of the cornea, with subtemporal thinning the most common (Romero-Jiménez et al., 2013; Sharif et al., 2018). The prevalence rate of KC is approximately 1/2000–1/500, and it usually occurs during puberty, generally in one eye, with early symptoms including blurred vision and photophobia. Visual acuity declines progressively as the disease progresses, with irregular corneal astigmatism, monocular diplopia, and even irreversible vision loss observed (Jones-Jordan et al., 2013; Mostovoy et al., 2018; Flockerzi et al., 2021). The etiology and causes of this disease have not yet been clarified; however, studies have suggested that it may be related to structural changes in the corneal collagen tissue (Kankariya et al., 2013). Early diagnosis of KC is difficult, and is generally made by comprehensive analysis of the corneal topography and biomechanical characteristics during evaluation (Randleman et al., 2008; Santodomingo-Rubido et al., 2022). Currently, contact lenses, corneal cross-linking treatment, keratoplasty, and several other methods are used to treat the disorder (Godefrooij et al., 2017; Röck et al., 2018; Ferdi et al., 2019); however, corneal transplantation can lead to rejection, complicated cataracts, iris atrophy, secondary glaucoma, and other problems (Shah et al., 2010). Therefore, the early detection of KC and timely intervention are of great significance in controlling the progress of the disease and maintaining good vision. AI models that are useful in diagnosing KC have so far been established using SVM, decision tree, CNN, multilayer perception neutral networks (MLPNN), and feed forward neural networks (FNN), all of which have been found helpful in the early diagnosis of KC (artificial intelligence and corneal diseases, 2022).

Combining corneal topography with AI was found useful in improving the accuracy of KC diagnosis. Using three types of convolution neural networks (ResNet152, VGG16Net, Inception v3), Kuo et al. (2020) constructed a DL model that can diagnose KC and 359 corneal topographic maps were used to train and verify the model. The results showed sensitivities and specificities of >0.90 for all the CNN models, of which ResNet152 exhibited the best diagnostic performance with an AUC value of 0.995. Al-Timemy et al. (2021) constructed an AI model that can diagnose KC based on the hybrid DL algorithm using 3,794 corneal images (divided into normal cornea, suspected KC, and keratoconus). According to data describing the anterior and posterior eccentricity, anterior and posterior sagittal arc, and corneal thickness, corneal features were extracted for training and verification of the AI model, with results indicating AUC values of 0.99 and 0.93 and accuracies of 0.988 and 0.815, respectively, for KC. Compared with the previous single CNN model, which is sometimes highly sensitive to slight disturbances in the pixels comprising the input image, the hybrid algorithm provides more reliable results. Zéboulon et al. (2020) established an ML model based on a CNN to diagnose KC. They collected 3,000 corneal topography maps (normal corneal topography, KC topography, and corneal topography with a history of refractive surgery) and used the data of anterior corneal height map, posterior corneal height map, anterior keratometry map, and corneal thickness map for training and testing. After testing, the results showed that the accuracy of the model was as high as 0.993 in diagnosing KC, and thus has potential for application in clinical practice. Kamiya et al. (2021) constructed a DL model for diagnosing normal corneas and KC based on the VGG-16 neural network, using 519 corneal topographic images that were coded by color to train and test the model. The results showed that the model performed well in diagnosing KC, with an accuracy of 0.966, sensitivity of 0.988, and specificity of 0.944. Using CNNs, Kato et al. (2021) constructed an AI model that could predict the progress of KC. In this study, 274 corneal tomography images were collected and before and after anterior keratogram and corneal thickness images combined to form the training set and test set of DL model. measured to form the training and test sets for the DL model. The results showed that AUC, sensitivity, and specificity values of 0.81, 0.78, and 0.70, respectively, for predicting KC progression. The above research results illustrate the usefulness of using AI for the auxiliary diagnosis of KC, for which it can significantly improve the accuracy of a diagnosis, save time, and provide the best treatment for patients.

AI can help ophthalmologists effectively distinguish KC from normal corneas and classify diseases by analyzing the corneal shape and thickness, among other parameters. In order to assist KC classification, Feng et al. (2021) created a DL algorithm (KerNet algorithm). They used 854 corneal images together with original data such as the anterior and posterior surface curvature, anterior and posterior surface topography, and corneal thickness to form a numerical matrix for the training and verification of the algorithm. The results showed that the algorithm could achieve better results than the most advanced methods in detecting and classifying KC, especially subclinical KC, with an accuracy of 0.95. To distinguish KC from subclinical KC and normal cornea, Abdelmotaal et al. (2020) constructed a DL model based on a CNN and collected 3,218 corneal images for training and testing. The model can be realized using a single image for highly accurate KC classification, with an average of 0.983, and the fact that less computing resources are required renders this model advantageous in terms of applicability to large-scale disease screening without sufficient data. Aatila et al. (2021) constructed some DL models using a variety of algorithms (random forest classifier, Gaussian naive Bayes classifier, K neighbors classifier, logistic regression, linear discriminant analysis, decision tree classifier, and support vector machine) for the classification of KC. They used 12 242 corneal topography maps to compare the classification performance of the different DL models. The results indicated that random forest had the best classification performance, with an accuracy as high as 0.95. Cao et al. (2020) constructed an AI model that can distinguish subclinical KC from non-KC based on a variety of DL algorithms (random forest, decision tree, logistic regression, support vector machine, linear discriminant analysis, multilayer perceptron neural network, lasso regression, and k-nearest neighbor). Corneal parameters of 49 subclinical KC and 39 control eyes were analyzed, with diagnostic results showing an AUC of 0.97 for the random forest random forest model. This indicates that selecting a combination of important parameters from a larger set of parameters would lead to more objective and effective KC screening when constructing a ML model, rendering it a useful tool in clinical practice. The above studies are summarized in Table 3.

**TABLE 3:** Application summary of different AI models and algorithms in keratoconus.

Authors	Task	Sample size	AI algorithms	Output
Kuo et al. (2020)	Diagnosis	359 images	ResNet152	AUC = 0.995
			VGG16Net	
			Inception v3	
Al-Timemy et al. (2021)	Diagnosis	3,794 images	unsupervised machine learning	AUC = 0.99, 0.93
				Accuracy = 0.988,0.815
Zéboulon et al. (2020)	Diagnosis	3,000 images	CNN	Accuracy = 0.993
Kamiya et al. (2021)	Diagnosis	519 images	VGG-16	Accuracy = 0.966, Sensitivity = 0.988, Specificity = 0.944
Kato et al. (2021)	Prediction	274 images	CNN	AUC = 0.81
				Sensitivity = 0.78
				Specificity = 0.70
Feng et al. (2021)	Classification	854 images	KerNet	Accuracy = 0.95
Abdelmotaal et al. (2020)	Detection	3,218 images	CNN	Accuracy = 0.983
Aatila et al. (2021)	Classification	12242 images	Random forest classifier	Accuracy = 0.95
			Gaussian naive bayes classifier	
			K neighbors classifier	
			Logistic regression	
			Linear discriminant analysis	
			Decision tree classifier	
			SVM	
Cao et al. (2020)	Classification	88 images	Random forest, Decision tree, Logistic regression, Support vector machine	Accuracy = 0.97
			Linear discriminant analysis	
			Multilayer perceptron neural network	
			Lasso regression	
			K-nearest neighbor	

Late keratoconus often leads to severe vision loss or even blindness in patients. Early and timely treatment can effectively alleviate the progression of keratoconus and protect patients’ vision. Therefore, for patients with keratoconus, early diagnosis and timely treatment are very important. The above AI studies show that AI has carried out a lot of research in the diagnosis, classification and prediction of keratoconus, and the AI model has shown good performance. AI model can provide great help to doctors in the clinical diagnosis of keratoconus, and automatically complete the relevant diagnosis and treatment work, so as to reduce the workload of doctors, which has important clinical significance to improve the efficiency of doctors.

### 2.4 Application of AI models and algorithms in the preoperative measurement and effect prediction of intraocular lenses

An intraocular lens (IOL) is a special lens made of synthetic materials that can replace the human lens (Lundström et al., 2018). The development of cataract surgery and the demand for high visual quality has meant that cataract surgery has evolved from visual rehabilitation to accurate refractive surgery with high visual quality (Piovella et al., 2019). The choice of intraocular lens has also gradually diversified from unifocal to functional intraocular lenses (Simon et al., 2014). The postoperative visual acuity of patients depends to a large extent on accurate biometric and IOL diopter calculations prior to operating. However, the changes that occur in the intraocular structure of some patients before surgery renders calculation of the IOL diopter difficult, and postoperative complications can easily occur in surgery for issues such as high myopia (Savini and Hoffer, 2018; Yao et al., 2021). In addition, the abnormal position of the IOL that can result from various factors will seriously affect visual quality after surgery (Wang et al., 2022). In recent years, IOL calculation methods based on AI have shown good performance and been proved able to effectively improve the accuracy of IOL diopter calculations (Wang et al., 2016; Melles et al., 2019; Nemeth et al., 2022). At the same time, a variety of methods such as slit-lamp photography, slit-lamp video photography, and OCT can be combined to determine the location of the IOL (Mura et al., 2010; Omoto et al., 2022).

Using AI in designing the IOL calculation formula can improve the accuracy of the calculation according to the characteristics of different patients so that better visual quality can be obtained following surgery. Mori et al. (2021) constructed an ML model based on a support vector regression algorithm to adapt the diopter calculation method for intraocular lens in a specific patient population to improve the accuracy of the calculation. By analyzing the clinical data for 11 611 eyes with a single monofocal IOL implantation model, the constants of the SRK/T and Haigis formulas were optimized and the support vector regression algorithm was used to adapt the SRK/T, Haigis, Hill-RBF, and Barrett Universal II formulas. The results showed a smaller average error in the optimized formula for calculating the IOL diopter than that obtained using the other formulas (*p* < 0.001). To improve the accuracy in calculating the IOL degree for high myopia, Wei et al. (2020) developed an AI calculation model based on the XGBoost regression algorithm. They collected data from 1,564 high myopia eyes for training and verification and combined a constant IOL diopter with the Barrett Universal II formula and other data, they developed a new IOL calculation method. The results showing significant decreases in the median absolute and median square errors as compared to those obtained under the BUII formula (*p* ≤ 0.001), and the proportion of eyes with prediction errors within ±0.25D increased significantly. Cabeza-Gil et al. (2020) proposed two intraocular lens calculation models based on the DNN algorithm to calculate the biomechanical stability of IOL which were then verified using data from 37,161 cases. Six parameters (length, width, thickness, opening angle of the haptic, tactility, and haptic-optic junction in the reference data) were used to enhance the consistency of patient characteristics so as to improve the success rate of the operation. The results showed Pearson’s r values of 0.995 and 0.992 for the two models; indicating good performance. Clarke and Kapelner (2020) designed a more accurate diopter calculation model for the intraocular lens, Bayesian Additive Regression Trees (BART), which was based on the ML algorithm. They collected measurement data from 5,331 eyes divided into training and verification subsets based on the specific characteristics of patients and their eyes. The results showed an average absolute error of 0.204 D, proving that the diopter of the intraocular lens calculated by this model was more accurate than that obtained by other commonly used formulas.

The calculation formula for the IOL has undergone several generations of evolution at different times. The first-generation formula is based only on regression data, while the second-generation formula includes the influencing factor of axis length and the third-generation formula refers to optics and IOL position factors. Methods for formula optimization are now more abundant, and combined with artificial intelligence, the accuracy of IOL calculation has been much improved. Guillaume et al. (2021) constructed an ML model based on the multiple linear regression algorithm to improve the IOL formula and constructed the new PEARL-DGS formula to calculate the diopter of IOLs by analyzing the data of 4,242 intraocular lens implants. The examination data of another 677 eyes were collected and compared with the K6 and Olsen, EVO 2.0, RBF 3.0, and BUII formulas, with results showing the smallest calculation error when using the PEARL-DGS formula with an error range of ±0.382 D, which indicates that completely retraining the formula, rather than the conventional constant adjustment, can allow adaption to the habits of doctors and the characteristics of specific patient groups. Ladas et al. (2021) constructed an AI model based on DL algorithms (extreme gradient boosting, support vector regression, artificial neural network) to optimize the existing diopter calculation formula for intraocular lens and develop a new hybrid formula based on AI. They analyzed the eye data of 1,391 patients who underwent IOL implantation, with the factor axial length, anterior chamber depth, lens thickness, sex, age, and postoperative significant diopter considered, and determined both the average absolute error in each IOL formula and the number of eyes that predicted diopter within 0.5 D. After AI optimization, the average percentage of eyes within ±0.5 D predicted by the SRK formula increased to 14%, the Holladay 1 formula increased by 9.3% and the LSF formula increased by 5.3% (*p* < 0.05), while in terms of average absolute error, the predicted diopter of optimized SRK formula decreased to 0.14 D, the Holladay 1 formula decreased to 0.08D and the LSF formula decreased to0.04D.

In the process of intraocular lens implantation, the influence that location and the size of the anterior and posterior space have on visual quality and postoperative complications can easily be ignored. An IOL localization method based on AI can effectively solve this problem. Schwarzenbacher et al. (2022) used a CNN to construct a DL model that can automatically divide the IOL, Retrolental, and Berger’s space and analyze the spatial resolution to accurately locate the target structure. In the study, a total of 92 eye OCT images were used to train and varify the model, with results indicating Precision, Recall, and Dice scores of 0.97, 0.90, and 0.93, respectively, indicating that the model has high accuracy in locating the IOL. This is the first time that this type of algorithm has been proposed to automatically segment the posterior structure of the anterior segment. To evaluate the position of the IOL in three-dimensional space, Xin et al. (2020) constructed an AI evaluation model based on a region-based fully convolutional network (R-FCN), with 86 AS-OCT images used to train and verify the model. The results showed an evaluation efficiency of 0.910 for the model, with intraclass correlation coefficients (ICC) of 0.867 and 0.901 for reliability and repeatability, respectively, evaluating the location of the IOL in a three-dimensional space to provide data support for the design of a better functional IOL. The above studies are summarized in Table 4.

**TABLE 4:** Application of different AI models and algorithms in intraocular lens calculation and postoperative prediction.

Authors	Task	Sample size	AI algorithms	Output
Mori et al. (2021)	Optimization	11611 eyes	Support vector regression	The average calculation error of IOL diopter calculated by the optimized formula is smaller than that of other formulas (*p* < 0.001)
Wei et al. (2020)	Design	1,564 eyes	XGBoost regression	Median absolute errors and median square errors decreased significantly (*p* < 0.001)
Cabeza-Gil et al. (2020)	Design	37161 individuals	DNN	Pearson’s r of two models = 0.995 and 0.992
Clarke and Kapelner (2020)	Design	5,331 eyes	Bayesian Additive Regression Trees	The average absolute error = 0.204 D
Debellemanière et al. (2021)	Optimization	4,919 eyes	Multiple linear regression	The error range = ± 0.382 D
Ladas et al. (2021)	Optimization	1,391 eyes	Support vector regression	The accuracy rate of each calculation formula is improved after optimization
			Extreme gradient boosting	
			ANN	
Schwarzenbacher et al. (2022)	Location	92 images	CNN	Precision = 0.97
				Recall = 0.90
				Dice score = 0.93
Xin et al. (2020)	Assessment	86 images	Region-based fully convolutional network	Intragroup correlation coefficient of reliability = 0.867
				Intragroup correlation coefficient of repeatability = 0.901

Intraocular lens implantation is a common method of ocular surgery. Patients’ postoperative visual acuity is highly correlated with their accurate preoperative biometric features and IOL diopter calculation. AI-based IOL diopter calculation can effectively improve the accuracy and help solve some cases with complicated intraocular structure. In addition, artificial intelligence can assist in determining the location of IOL implantation, which plays an important role in improving patients’ visual quality and reducing postoperative complications.

### 2.5 Application of AI models and algorithms in amblyopia

Amblyopia is a decrease in monocular or binocular best-corrected visual acuity that leads to abnormal visual experiences (monocular strabismus, anisometropia, high ametropia, and form deprivation) during visual development (Kates and Beal, 2021). Factors that cause amblyopia include ametropia, strabismus, anisometropia, ptosis, lens opacity, and form deprivation (Paff et al., 2010; Rajavi et al., 2012; Barrett et al., 2013). According to its etiology, amblyopia is mainly divided into strabismic, anisometropic, ametropic, and form deprivation amblyopia (Maurer and K. S., 2018; Birch, 2013). Amblyopia can be mild, moderate, or severe. The main manifestations of amblyopia are lower than normal best-corrected visual acuity, crowding, paracentric fixation, prolonged PVEP latency, and decreased amplitude of visual evoked potentials (Lempert, 2006; Hess and Thompson, 2015). At present, the main treatment strategy for amblyopia is to remove the factors that cause deprivation as soon as possible, with cataract treatment, complete ptosis correction, the use of appropriate corrective glasses, covering healthy eyes, and optical drug suppression therapy all used (Birch et al., 2021; Boniquet-Sanchez and Sabater-Cruz, 2021; Meier and Tarczy-Hornoch, 2022). According to the law of visual development, early detection, diagnosis, and intervention are particularly important for patients with amblyopia, and can significantly improve the therapeutic effects (Holmes and Levi, 2018).

Murali et al. (2020) constructed a DL model based on a CNN for screening the risk factors for amblyopia in children. This model can identify biological characteristics such as corneal light reflection, iris center position, pupil radius, and ratio of eye radius to iris diameter from the facial image, allowing easy screening of the risk factors in children. They collected facial images of 54 participants to train and test the model, with results indicating an accuracy of 0.796, sensitivity of 0.882, specificity of 0.756, and an F-score of 0.732. Murali et al. (2021) collected facial images of 654 participants (randomly divided into training and verification sets) and constructed a DL model that could screen and identify the risk factors of amblyopia in children based on a convolution neural network. After verification, the values of 0.908, 0.836, and 0.859, respectively, for accuracy, sensitivity, and specificity indicate that the use of DL to analyze photographic images is an effective alternative method for screening risk factors in children with amblyopia. The above studies show that using AI to recognize the biological features of children’s facial images allows accurate detection of the risk factors for amblyopia, which is of great significance for amblyopic children. The above studies are summarized in Table 5.

**TABLE 5:** Application summary of different AI models and algorithms in amblyopia.

Authors	Task	Sample size	AI algorithms	Output
Murali et al. (2020)	Detection	54 images	CNN	Accuracy = 0.796
				Sensitivity = 0.882
				Specificity = 0.756
				F-Score = 0.732
Murali et al. (2021)	Detection	654 images	CNN	Accuracy = 0.908
				Sensitivity = 0.836
				Specificity = 0.945
				F-Score = 0.859

Amblyopia is a common eye disease in children. for amblyopic children, the therapeutic effect is closely related to age, and the younger the age, the better the therapeutic effect. In addition, early treatment not only has a short course of treatment, but also has a significantly higher cure rate. Therefore, it is particularly important to screen the risk factors of amblyopia in children. Through the above research, we can see that AI shows a good performance in the screening of risk factors of amblyopia in children. Its use in the screening of risk factors of amblyopia in children can not only save manpower, material and financial resources, but also is of great significance for the early treatment of children with amblyopia.

## 3 Limitations and challenges

As can be seen from the above studies, AI has been widely used in optometry. Many AI models and algorithms have shown superior performance in the diagnosis, identification, screening, prediction, and treatment of disease with satisfactory results achieved. However, there remain many challenges and limitations that are likely to seriously affect further research and the application of AI in the field of optometry. For example, 1) The quality of the image in the data set (Ting et al., 2019; Xie et al., 2020). The datasets used in some studies are public, and include many poor-quality images. Because the research results of the AI model are closely related to image quality, this issue will significantly impact the AI model, resulting in inaccurate results. 2) Sample size (Chen et al., 2018; Huang et al., 2021; Gutierrez et al., 2022; Huang et al., 2022). The small sample size in some studies is likely to affect the stability of the AI model, affecting the reliability of the results. For example, in some AI studies of strabismus, amblyopia and other diseases, the sample size in the data set is small, which will have a certain impact on the performance of the final AI model. 3) External verification of the algorithm (Wawer Matos et al., 2022; Wong et al., 2022). Some AI models have excellent performance in training and verification; however, there is a huge gap between the “real environment” and the “research environment”, which may lead to performance degradation and produce unstable results when such AI models are applied to clinical diagnosis and treatment. For example, in the research of strabismus, keratoconus and other diseases, many AI models are verified only on external data sets, but not in the “real environment.” 4) Validity of the datasets (Ting et al., 2019; Ng et al., 2021). The images used in many studies need to be annotated, with strict requirements for labeling l. The validity of the data used is particularly important for the research results of an AI model. For example, in some studies, the task of image annotation is completed by residents, which may be difficult to ensure the accuracy of image annotation, thus affecting the performance of the AI model. 5) Interpretability of AI algorithms (Al-Aswad et al., 2022; Betzler et al., 2022). Because AI belongs to a subfield of computer science, many clinical medical staff have little AI-related knowledge, which leads to incorrect interpretation in the process of clinical application, resulting in the so-called “black box phenomenon.” 6) AI model may lead to medical legal problems (Ji et al., 2022). No one is perfect, and artificial intelligence cannot be 100% accurate. When the diagnosis of the AI model is wrong or even has serious consequences, how to determine its behavior? Who should bear the consequences? These will become some thorny medical legal issues. 7) The privacy and security of patients (Murdoch, 2021). Medical data focus on patients’ health, disease status, biological genes and other information, once leaked, the consequences are unimaginable. The privacy security problems of medical artificial intelligence are as follows: do patients fully get their informed consent in the process of data collection? In the event of a privacy leak, who will be held responsible? Who has the right to get information about a patient’s health or disease? These are all important and urgent problems to be solved. 8) The lack of legal protection related to AI technology. In recent years, the rapid development of AI technology has greatly changed our lives. However, the legislative process for artificial intelligence is relatively slow. Artificial intelligence needs to have corresponding laws and regulations in all aspects of research and development, development and production process, and stipulate the ownership of responsibility and the direction of development; artificial intelligence (especially deep learning) if there is no legal escort, will seriously affect its development and application.

## 4 Conclusion

Through the above AI research, it can be found that many research achievements have been made in the application of AI in the field of optics, and the application prospect is very broad, which can bring reform and progress to the field of optics in many aspects. Intelligent systems based on different AI algorithms can help ophthalmologists better diagnose and treat diseases in the field of optometry according to patients’ eye clinical data and personal data, which has important clinical significance. But for clinical medical staff, only this is far from enough. Because this shallow clinical application plays a more auxiliary role, such as reducing the repetitive physical labor of clinical medical personnel, improving the accuracy of diagnosis and so on. If we want to fully apply AI to ophthalmic clinic, AI must have more functions. It can not only complete the assigned tasks, but also develop more technologies and methods according to the characteristics of each task. In addition, AI also needs to pay more attention to those unsolved technologies and challenges, so as to better promote the clinical application of AI in ophthalmology.

As mentioned in this review, AI can complete specified tasks by building algorithm models and DL networks, particularly image recognition, classification, diagnosis, and data analysis. Although there are still several challenges associated with AI modeling, it can provide doctors with objective clinical decisions, laying the foundation for accurate treatment. There is an urgent need for future research into the unknown aspects of target diseases, which combined with application, could instigate targeted and high-quality research. Simultaneously, the introduction and application of a number of standardized norms are that can further improve the quality of AI medical research and promote AI products will provide great advantages in the diagnosis and treatment of optometry-related diseases as soon as possible.

## 5 Resource identification initiative

PubMed (RRID:SCR_004846).
